# Single-Stoma Cutaneous Ureterostomy After Radical Cystectomy: A Contemporary Narrative Review

**DOI:** 10.3390/curroncol33080455

**Published:** 2026-07-29

**Authors:** Raymundo A. Munoz, Luis G. Medina, Jonathan S. Kim, Allison Supernaw, Matvey Tsivian

**Affiliations:** 1 Division of Urology, Department of Surgery, University of Colorado Anschutz School of Medicine, Aurora, CO 80045, USA; raymundo.munoz@cuanschutz.edu; 2 Department of Urology, Medical University of South Carolina, Charleston, SC 29425, USA; medinana@musc.edu (L.G.M.); allison.supernaw@sec.edu (A.S.); 3 Department of Urology, Emerson Health Hospital, Concord, MA 01742, USA; jsk333@drexel.edu

**Keywords:** cutaneous ureterostomy, urinary diversion, radical cystectomy, ureterocutaneostomy, narrative review

## Abstract

Radical cystectomy (RC) with urinary diversion (UD) is the standard treatment for muscle-invasive bladder cancer. Although the ileal conduit (IC) is the most commonly performed diversion, its reliance on bowel reconstruction leads to substantial perioperative morbidity and long-term complications. Cutaneous ureterostomy (CU) has re-emerged as an attractive alternative, particularly for elderly and medically frail patients, due to its technical simplicity and avoidance of intestinal manipulation. Recent single-stoma and tubeless modifications have aimed to overcome historical limitations of CU, including stomal stenosis and long-term stent dependence. Compared with IC, modern refinements of single-stoma CU showed shorter operative times and generally shorter hospital stays, largely reflecting avoidance of bowel reconstruction. Estimated blood loss and overall intraoperative complication rates were broadly comparable between diversion types. Contemporary retrospective data on single-stoma modifications showed lower rates of stomal stenosis and improved catheter-free outcomes compared with historical data, while infectious complications and readmission rates remained similar to those of IC in most series. Renal outcomes generally remained stable, with patient factors such as baseline renal function, hydronephrosis, and stent dependence appearing to be more influential for long-term deterioration than diversion type alone. Quality of life after contemporary single-stoma CU was similar to IC, based on the available retrospective evidence. Overall, contemporary single-stoma CU represents a valuable bowel-sparing urinary diversion that may potentially reduce operative burden while maintaining acceptable functional and quality-of-life outcomes. Current retrospective evidence supports its role as a particularly attractive option for elderly and frail patients, although prospective, multicenter comparative studies with standardized outcome reporting are still needed to better define patient selection and long-term outcomes.

## Simple Summary

Radical cystectomy and urinary diversion after neoadjuvant therapy is the gold standard treatment for muscle-invasive bladder cancer. Ileal conduit remains the most commonly used diversion as it is associated with fewer complications when compared to other continent urinary diversions; however, it requires bowel reconstruction and may be associated with significant morbidity. Single-stoma cutaneous ureterostomy is a simpler alternative that avoids bowel manipulation and may be particularly attractive for older or medically frail patients. Historically, single-stoma cutaneous ureterostomies have been associated with poor outcomes, yet recent technical refinements aimed at improving outcomes have renewed interest in this approach. In this review, we summarize current evidence on contemporary single-stoma cutaneous ureterostomy, including its history, surgical techniques, perioperative outcomes, complications, long-term outcomes, and quality of life. By synthesizing the available literature, this review clarifies the role of cutaneous ureterostomy in contemporary radical cystectomy and highlights areas where additional prospective research could be directed.

## 1. Introduction

Bladder cancer ranks as the tenth most prevalent cancer globally, with its incidence increasing worldwide [1]. Neoadjuvant therapy followed by radical cystectomy (RC) and urinary diversion (UD) is considered the standard treatment for muscle-invasive bladder cancer according to the NCCN and AUA/ASCO/SUO guidelines [1,2,3]. The choice of diversion technique remains one of the most consequential decisions impacting perioperative morbidity, renal function, and quality of life [1,4]. UDs vary in physiological, technical, and functional traits and are broadly classified into three categories: incontinent, continent cutaneous (e.g., Indiana and Charleston pouches), and continent orthotopic neobladder diversions (ONB) (e.g., Studer neobladder) [1,4].

Among the available diversion options, the ileal conduit (IC) remains the most commonly performed technique. First described by Bricker in 1950, the IC is created by connecting the ureters to an isolated distal ileum segment, which is then brought to the abdominal wall to form a stoma [5]. Several factors contribute to the widespread preference for IC, including its reproducibility, broad applicability across age groups and comorbid conditions, and decades of refinement that have led to standardized stomal and ureteroenteric anastomotic techniques [1,4]. Despite these benefits, IC has notable perioperative and long-term complications, mainly concerning bowel anastomosis and chronic exposure of the ileal segment to urine [6,7]. Reported complication rates within 90 days range from 50–70%, with major (grade III-V) complications in roughly 20–30% of cases [8]. Long-term sequelae include parastomal and incisional hernias, ureteroenteric strictures, and urolithiasis, among others [1,4]. Collectively, the morbidity linked to IC has renewed interest in bowel-sparing diversion techniques.

Cutaneous ureterostomies (CU) have emerged as an important form of incontinent UD, offering a less invasive alternative to more complex reconstructive procedures. This technique involves directly bringing one or both ureters to the abdominal surface to create a single stoma for continuous urine drainage [4]. Notably, CU does not require intestinal reconstruction, which has led to suggestions that it can reduce operative times, blood loss, and complication rates [4]. Its technical simplicity has significant implications for perioperative outcomes, especially in elderly, malnourished, and medically complex patients, where longer surgeries and significant physiological stress could increase morbidity. Propensity-score data also indicate that selecting among UD types (particularly IC and CU) may not affect key oncological milestones such as 2-year overall, cancer-specific, and recurrence-free survival rates [9]. This suggests that the type of diversion itself may not directly influence oncological outcomes but rather reflects the characteristics of the population undergoing RC. However, current treatment guidelines for non-metastatic muscle-invasive bladder cancer from the AUA, ASCO, ASTRO, and SUO are vague regarding CU’s role as a UD option after RC [10].

Recent advances in CU surgery include single-stoma bilateral routes, which streamline care and lessen appliance burden compared to double-stoma setups [11]. Modified tubeless techniques aim to reduce dependence on indwelling stents by improving uretero-cutaneous anastomosis through spatulation, anti-reflux measures, and stomal maturation [11]. These refinements reflect an aim to mitigate the limitations of CU and further optimize this diversion for contemporary practice.

Despite these benefits, CU still has limitations and potential complications. Intraoperative limitations must be considered when performing CU, including ureteral length and tension, compromise of the ureteric blood supply, anatomical variations, and abdominal wall thickness, all of which can limit CU’s feasibility [1,12,13]. Long-term concerns are also highly relevant and include increased risks of UTIs and ureteric strictures, which may lead to frequent stent exchanges and declining renal function [14,15]. Reported stomal stenosis rates vary widely; recent studies using modified Toyoda or single-stoma tubeless techniques report rates of about 9–11%, whereas unmodified cohorts have shown rates of 47–70% [16,17]. Furthermore, in propensity score-matched comparisons with IC, recurrent pyelonephritis was seen in up to 25% of CU patients [18]. Understanding these complications in the context of their modifiable risk factors is crucial for providing preventive intervention and risk stratification.

The recognition that outcomes can be improved over time through technique optimization and perioperative strategies suggests that historical comparisons may underestimate CU’s potential when current best practices are applied. Since most patients undergoing RC are over 65, have ASA scores ≥ 2, and have high comorbidity burdens, these factors may influence the choice of UD and its outcomes, particularly when considering the role of CU in frail or high-risk patients [19,20].

This comprehensive review examines the current literature on single-stoma CU, focusing on various aspects of this surgical approach and its clinical implications. We analyze surgical techniques and modifications, evaluate perioperative and long-term outcomes in recent series, describe procedure-related complications, identify risk factors for specific issues, and compare CU outcomes to alternative UD methods. By synthesizing emerging data, this review aims to clarify CU’s role in modern UD decision-making and highlight areas for future prospective research.

## 2. Materials and Methods

**Figure 1:**
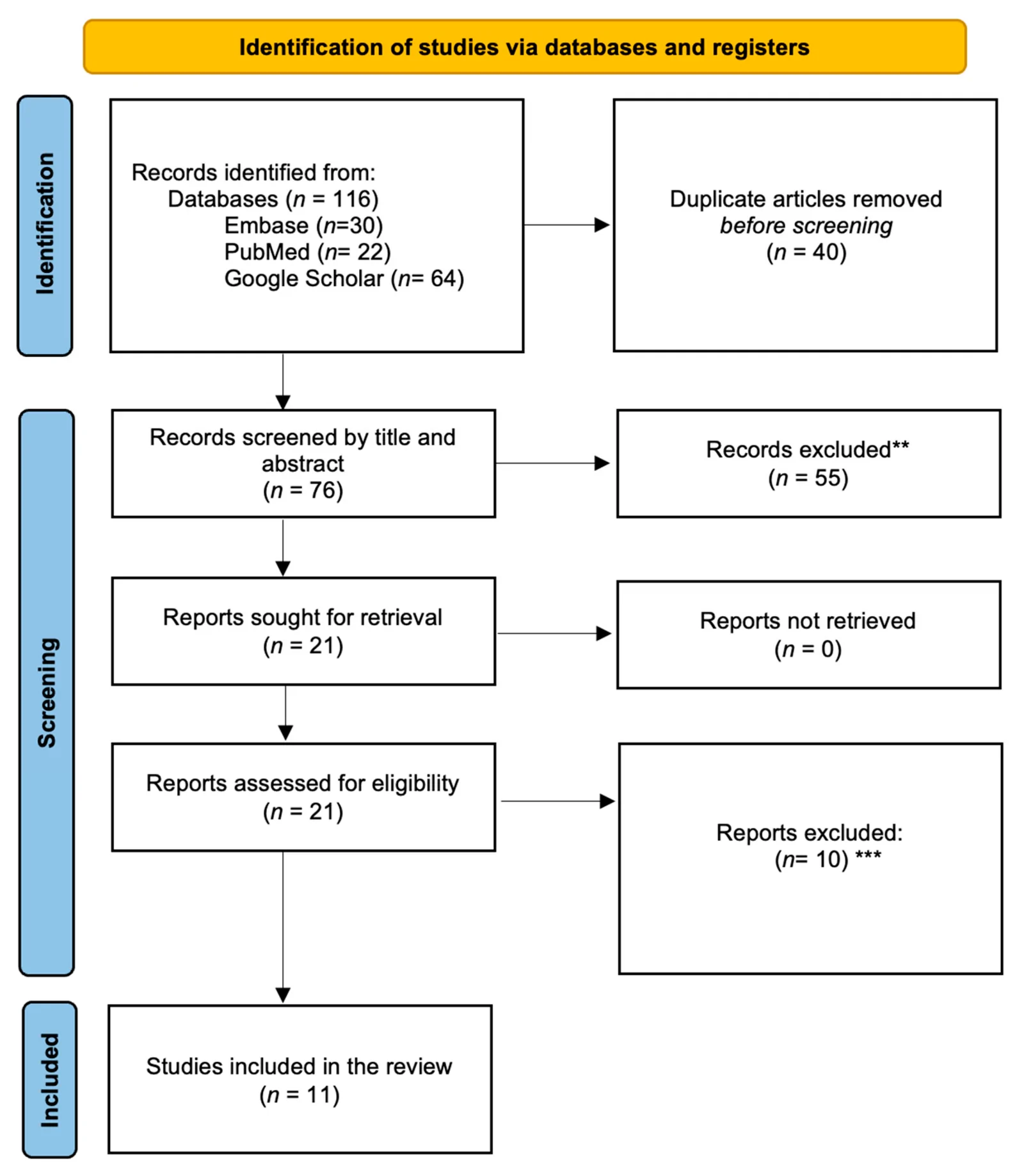
Study Selection Flow diagram. ** Records excluded by exclusion criteria: duplicates and out-of-scope studies. *** Records excluded by exclusion criteria: outcomes differ, and non-English articles, studies that did not specify whether a single or double stoma was used, or that did not present individual data for each urinary diversion technique.

This manuscript presents a narrative, non-systematic literature review compiled from articles retrieved using PubMed, Embase, and Google Scholar databases. The literature search was performed in March 2026, using MeSH terms and keywords, including “ureterostomy,” “single-stoma cutaneous ureterostomy,” “ureterocutaneostomy,” “cystectomy,” “radical cystectomy,” and “urinary diversion.” The search was limited to studies published between January 2015 and March 2026 to capture contemporary evidence on single-stoma cutaneous ureterostomy (CU). Non-English publications and abstracts without full text were excluded. The reference lists of selected articles were reviewed to find additional studies. Only peer-reviewed original studies reporting single-stoma CU data were included; studies that did not specify whether a single or double stoma was used, or that did not present individual data for each urinary diversion technique, were excluded. We included all studies regardless of sample size, study design, or citation significance. The initial search yielded 116 articles in total, and the search strategy is outlined in Figure 1. Two paired investigators (R.A.M. and L.G.M.) independently screened for eligibility. Following full-text review, 11 studies were selected for final inclusion [8,11,21,22,23,24,25,26,27,28,29].

## 3. Results

Out of the 11 studies included, more than two-thirds were retrospective, and none employed propensity score matching. Notably, one-third of the studies originated from the same institutions and research groups (Tsaturyan & Arman; both Thakker et al. articles), with some overlap in patient populations [8,22,23,27]. Only three studies were multicentric, and nine were comparative in design. Detailed characteristics and data from the included studies are shown in Table 1.

## 4. Historical Overview of CU Progression and Development

CU was first described by John Simon in 1852 while treating a child with bladder exstrophy [30]. Early on, it showed considerable limitations, as up to two-thirds of patients developed stomal stenosis or recurrent UTIs, often requiring long-term ureter stenting [31]. Despite not requiring bowel manipulation, the added morbidity and dual stomas made it less appealing, leading to the ileal conduit (IC) or orthotopic neobladder diversions (ONB) becoming the gold standard options for urinary diversion (UD) [1]. However, it remained an option for palliative care and has been continually revisited and modified to reduce morbidity (Table 2) [32,33].

In 1975, Ariyoshi et al. introduced the first major modification, creating a ureteral nipple with a triangular skin flap to reduce strictures, successfully achieving a primary tubeless nipple stoma in 61.9% of 21 patients [34]. In 1976, Toyoda et al. proposed a modification to improve ureter–skin fixation by creating a longitudinal ‘fish-mouth’ opening on the ureter, suturing it to the skin, and forming a double-barrel single-stoma with both ureters incised and sutured separately [35]. His technique achieved a catheter-free rate of 76% among 97 patients, as reported by Terai et al. [36]. Furthermore, the catheter-free rate remained stable over 5 years, ranging from 71% to 77% [36].

Various modifications to initial techniques have continually been proposed over time, such as the use of a skin flap with transverse nephropexy to reduce ureteral tension and maintain vascular supply (Kerney Modification), a ‘butterfly’ spatulation of the ureters with omental wrapping (Bolzano Technique), or fixation of the ureter to the rectus sheath (Okada modification) [37,38,39]. The Okada modification, in particular, focuses on stabilizing the abdominal tunnel to reduce the need for permanent stents; in 54 patients (102 renal units), this modification was associated with an improved catheter-free rate, from 60.5% (26/43 renal units) without stabilization to 89.8% (53/59 renal units) with stabilization [39]. In 2019, Tsaturyan et al. introduced one of the newest modifications by preserving the parietal peritoneum, creating a single oval stoma by anastomosing both ureters side by side on a single plate and fixing them to the abdominal wall, resulting in a catheter-free rate rising from 42.3% to 76.9% (OR = 5.13, 95%, 1.42–18.55) [22].

## 5. Intraoperative and Early Postoperative Outcomes

RC is a complex procedure associated with significant perioperative morbidity regardless of surgical approach or UD. Operative times typically range from 4–7 h, estimated blood loss (EBL) from 300–800 mL depending on approach, intraoperative complication rates from 1–7%, and length of hospital stays (LOS) from 4–10 days [40]. Minimally invasive procedures tend to take longer than open surgery; robot-assisted radical cystectomy (RARC) typically lasts about 76 min longer than open surgery (ORC) (39–112, *p* < 0.001) [40], while laparoscopic radical cystectomy (LRC) also takes more time than ORC (282 ± 51 min. vs. 235 ± 31 min., *p* < 0.001) [41]. The type of UD also influences operative duration, as ONB reconstruction is usually longer (434 ± 102.9 min) than IC (316.5 ± 72.4 min) and CU (262.7 ± 77.9 min) [42]. In contrast, ORC is associated with greater EBL than RARC (322 mL more) and LRC (295 mL more), as well as higher transfusion rates than RARC (OR 2.3, 95% CI 1.65–3.36, *p* < 0.001) [33,34,40,41]. Despite differences in operative techniques, overall intraoperative complication rates are comparable across techniques [41,43]. Although ERAS protocols have improved recovery and reduced overall LOS, the additional benefit observed with RARC remains modest (0.21 days, *p* = 0.02) [40,44].

### 5.1. Operative Time

Among single-stoma CU studies, this diversion method showed shorter operative times than IC, with mean durations ranging from 3–3.5 h for CU versus approximately 4.5–5 h for IC, despite variations in reporting methods [8,11,21,22,24,25,27]. This likely reflects the absence of bowel resection and reconstruction, which simplifies the procedure and leads to shorter operative times aside from the RC time [9]. Furthermore, newer single-stoma refinements such as the Yerevan and Li modifications achieved operative times comparable to their traditional counterparts (Ariyoshi: 194.3 ± 24.7 vs. Yerevan: 201.7 ± 23.2; Toyoda: 222.3 ± 36.3 vs. Li: 233.1 ± 29.7), suggesting that efforts to reduce stomal complications may not necessarily increase operative duration [22,25].

A shorter operative time may be one mechanism by which CU reduces perioperative morbidity. In a cohort of 296 patients undergoing RC with UD, Hanna et al. found that prolonged procedures were associated with higher rates of major complications (Clavien-Dindo ≥ III) after multivariable adjustment (OR 2.340, 1.288–4.250, *p* = 0.005), delayed bowel recovery (54.2% vs. 30.8%, *p* = 0.001), and increased 30- and 90-day readmissions (OR 2.617, 1.503–4.559, *p* = 0.001) [45]. These observations have been supported by large NSQIP analyses, in which each additional hour of operative time independently increased risks of thromboembolic events, UTI, transfusion, readmission, and overall postoperative complications (ORs 1.04–1.23) [46,47]. Haeuser et al. observed that this effect was particularly strong in older patients and those with ASA scores > 3; these groups had 1.01- and 1.29-fold higher odds of major complications, respectively, and often overlap with potential CU candidates [47].

It should also be noted that minimally invasive RC, particularly robot-assisted radical cystectomy (RARC), is associated with longer operative times, as demonstrated in randomized clinical trials such as RAZOR (7.1 h [5.3–8.4] vs. 6.0 h [4.6–7.5]; *p* = 0.0005), as well as in laparoscopic radical cystectomy (LRC) versus open surgery (ORC) comparisons [41,43]. Despite this increase in operative duration, both robotic and laparoscopic approaches have consistently shown comparable trans- and perioperative outcomes to open surgery in randomized trials and meta-analyses, supporting their safety [40]. This apparent discrepancy highlights that operative time alone is not the sole determinant of perioperative outcomes and that, in fact, it should also be contextualized into the approach employed for RC [48].

Overall, the mean operative duration was 1–2 h shorter for single-stoma CU than for IC across the studies analyzed, given that different approaches may vary in overall RC time. These findings highlight that operative time is a modifiable factor during RC that should be minimized, when possible, especially in older and comorbid patients, for whom minimizing surgical stress is particularly important.

### 5.2. Intraoperative Complications

Intraoperative urological complications are notoriously underreported and inconsistently documented. For RC, the reported incidence ranges widely in the literature from 1% to 88% [49,50,51]. When reported, most complications involve vascular (41–81.5%), gastrointestinal (3–17%), or urinary tract injuries (2–3%) [49,50].

Although randomized trials suggest that the surgical approach does not significantly affect intraoperative complication rates, an important question is whether the type of UD influences these complications. However, comparative data evaluating intraoperative complications by UD techniques are limited. In one cohort of 70 patients (35 CU vs. 35 IC), CU was associated with fewer intraoperative complications (5.7% vs. 25.7%, *p* = 0.04), while rates of hypotension (5.7% vs. 14.2%, *p* = 0.42), oxygen desaturation (2.8% vs. 11.4%, *p* = 0.35), arrhythmia (2.8% vs. 5.7%, *p* = 1.00), and bleeding (2.8% vs. 8.5%, *p* = 0.60) were comparable between groups [21].

In summary, the existing body of evidence does not show a clear difference in the type or severity of intraoperative complications between single-stoma CU and IC. Overall, these complications appear to be influenced mainly by RC complexity rather than by the choice of UD [51].

### 5.3. Estimated Blood Loss

Minimizing estimated blood loss (EBL) is important because its consequences extend beyond the immediate perioperative period. In a propensity score-matched study, Kamei et al. identified blood loss as an independent predictor of surgical site infection (OR 1.13 per 100 mL increase, 1.02–1.25, *p* = 0.001), with volumes exceeding 1630 mL associated with particularly high risk [52]. Beyond perioperative morbidity, excessive bleeding may also adversely affect oncologic outcomes. In 722 patients, blood transfusions independently predicted worse cancer-specific survival after adjustment, with lower survival in those receiving intraoperative (48 vs. 67%, *p* < 0.001) or postoperative transfusions (48 vs. 63%, *p* < 0.001) [53]. Notably, older age and greater comorbidity burden (ASA ≥ 2) are associated with increased intraoperative bleeding, suggesting that efforts to minimize blood loss may be especially relevant in these higher-risk populations [54].

Overall, available retrospective data reported EBL comparable between CU and IC, with ranges of 200–450 mL for CU and 400–500 mL for IC [21,22,23,25]. Longo et al. found significantly lower transfusion rates (17.1% vs. 42.8%, *p* = 0.03) and EBL in CU patients compared to IC (380 ± 93 mL vs. 510.5 ± 106.8 mL, *p* < 0.001) [21]. However, these results should be interpreted cautiously due to the retrospective design and small sample size, as most studies show no significant difference in intraoperative transfusion rates between CU and IC [22,24,29]. Since differences in EBL and transfusion rates among UD types appear modest, the surgical approach likely plays a larger role in bleeding outcomes than the diversion itself, with randomized trials consistently showing lower EBL and transfusion requirements for RARC and LRC compared with ORC [41,43,55].

### 5.4. Length of Hospital Stay

Length of stay (LOS) was found to be generally shorter after CU than IC across most series, with reported LOS ranging from 3–16 days for single-stoma CU versus 4–20 days for IC [8,21,24,25,27,29]. This difference could reflect faster postoperative recovery, likely due to the absence of bowel reconstruction and its effects on recovery and gastrointestinal-related complications, as observed in propensity-score studies comparing CU and IC [9]. Consistent with this, CU was associated with earlier drain removal (3.7 ± 0.9 vs. 6.2 ± 2.4 days, *p* < 0.01) and resumption of oral diet (2.0 ± 1.1 vs. 3.6 ± 2.7 days, *p* < 0.01) [21,29]; this aligns with a Mayo Clinic series of RARC with CU, in which flatus passage and diet resumption occurred early after surgery, contributing to a median LOS of only 3–4 days [26].

Perioperative care pathways also play an important role in postoperative recovery after RC. ERAS protocols have consistently been associated with faster gastrointestinal recovery, reduced morbidity, and shorter hospitalization without increasing mortality [44]. A meta-analysis including 4048 patients demonstrated a mean reduction in LOS of 4.54 days following ERAS implementation (−5.79 to −3.28, *p* < 0.001) [56,57], while earlier tolerance of solid food and earlier return of bowel function have also been consistently reported [57]. Among the reviewed studies, only da Costa et al. incorporated an ERAS protocol and still reported a significantly shorter LOS with CU than IC (13 ± 8.9 vs. 20 ± 14.5 days, *p* < 0.01) [29]. In this context, the advantages of ERAS and CU may be complementary, as avoidance of bowel reconstruction may further facilitate the accelerated recovery and discharge goals promoted by ERAS pathways [26,58,59].

The surgical approach has also been shown to play a significant role in overall LOS. A meta-analysis of randomized controlled trials by Khetrepal et al. indicates that LOS is shorter for RARC than for OR, with a mean difference of 0.21 [40]. However, Kadoriki et al. found in their propensity-score study of patients undergoing RARC and CU or CI that, after matching their cohorts, LOS was 2 days shorter, with better gastrointestinal outcomes as the main drivers of their results [9]. Since a higher comorbidity burden, including elevated Charlson Comorbidity Index scores, has been associated with longer LOS after RC (1.64 additional days per point increase; *p* < 0.001) [56], underscoring the importance of strategies that facilitate recovery and reduce hospitalization could be of particular value in patients with limited physiologic reserve. Therefore, the 1–4-day difference in LOS observed across the reviewed studies may be particularly relevant for patients undergoing RC, especially given the common frailty of this population.

## 6. Postoperative Outcomes

RC remains associated with significant postoperative morbidity regardless of UD type, with complication rates reported between 28–68% in recent systematic reviews and readmission rates ranging from 18–39% at 30 days and 17–58.5% at 90 days [20,32,60].

Although CU offers several perioperative benefits, it also presents unique challenges, including stomal stenosis, the need for upper-tract instrumentation, infectious complications, and long-term renal function decline. Historical CU series report stomal stenosis rates of up to 69% and infection rates of up to 44% [32,60]. Readmission rates reach approximately 22% and 40% at 30 and 90 days, respectively, with infections representing a common cause of rehospitalization [61]. Other reported complications include urolithiasis (4–40%) and progressive renal function decline [15,25,28,62,63]. However, interpretation of these outcomes requires caution, as patients undergoing CU are often older, frailer, and have worse baseline renal function, potentially introducing substantial selection bias when comparing diversion types [64]. Consequently, some adverse outcomes attributed to CU may partially reflect patient baseline characteristics rather than the diversion itself. The upcoming subsections will examine evidence regarding the main complications of CU, including stomal stenosis, infections, readmissions, other complications, and renal function following single-stoma CU.

### 6.1. Stomal Stenosis

Stomal stenosis is a common and significant complication after CU, affecting up to 69% of patients in early historical data and requiring long-term stenting with frequent exchanges to prevent full stomal closure [1,61]. It occurs secondary to distal ureteral ischemia, poor ureter–skin anastomosis, or tension at the suture line, resulting in granulation tissue and scarring [65]. Technical modifications aimed at improving ureteral vascularity and reducing anastomotic tension have substantially lowered stenosis rates, with the Toyoda technique and Okada modification reporting rates of 11–18% and 12%, respectively [17,36,39]. Modern refinements have further improved outcomes, as recent data from the Mayo Clinic report a 6.4% stenosis rate at 1.2 years and a 3.2% stoma ischemia rate at 30 days [26]. Reflecting these technical improvements, stenosis rates in contemporary single-stoma CU series ranged from 1% to 29%, although comparisons with IC have been mixed [8,11,22,25,27,28,29]. Da Costa et al. reported similar rates for CU and IC (1.8% vs. 5.7%, *p* = 0.3), whereas Zhang et al. reported higher rates in CU (29.2% vs. 11.8%, *p* = 0.01) [28,29]. However, factors beyond UD type may also affect stenosis rates, as Zhang et al. found that postoperative BMI changes influenced rates, with incidence decreasing from 45.5% to 36.8% in patients who experienced weight loss [28].

The development of stomal stenosis is closely linked to the need for ureteral stenting, making stent-free status an important outcome after CU. The need for long-term maintenance to maintain anastomosis patency has been a point of concern for CU; some series report long-term stent-free rates exceeding 70%, whereas others indicate that up to 88% of patients require temporary or permanent stenting to maintain stomal patency [9,36]. In our practice, stents are routinely removed before discharge. Modern single-stoma CU modifications seek to reduce both stenosis and stent dependency by improving ureteral alignment, minimizing tension, and preserving blood supply. Consistent with this, Li et al.’s modification of the Toyoda technique showed a reduced stenosis rate in their series (12.2% vs. 37.5%, *p* = 0.01) along with increased stent-free rates (90.8% vs. 71.8%)—the surgical modification was found to be an independent predictor of stent insertion (HR = 0.268, *p* = 0.001) [25]. Similarly, Thakker et al. reported a 17% stenosis rate with a modified Ariyoshi technique and a 74% stent-free rate at 12 months; in a subsequent comparative study, CU and IC showed similar rates of stent replacement (32% vs. 19%; *p* = 0.3) and stenosis (5% vs. 0%, *p* = 0.4) [8,27].

Stomal stenosis will remain a concern after CU, requiring surveillance and sometimes secondary interventions. Despite this, modern refinements have lowered stenosis rates from 69% in historical data to 1–29% with contemporary improvements. Improved stent-free outcomes indicate that this complication is becoming more manageable and technique-dependent, with current retrospective data showing rates as high as 90%.

### 6.2. Infections

Historically, CU was thought to carry a higher risk of infection and a greater need for antibiotic treatment than bowel-based diversions, due to the absence of anti-reflux mechanisms and the direct exposure of the upper urinary tract to the outside [32,64]. However, contemporary single-stoma series report pyelonephritis rates ranging from 7–22% at 30 days and 17–33% at 90 days [8,27,29], with infectious outcomes generally comparable to IC [8,21,28,29]. Contemporary series reported lower rates of pyelonephritis with their modified techniques compared with the traditional Toyoda (12.2% vs. 32.5%, *p* = 0.03) and Ariyoshi (13.3% vs. 23.3%) methods [16,19].

Long-term stenting and repeated urinary tract manipulation have been associated with an increased risk of infection [66,67], and Li et al. reported higher rates of pyelonephritis among stented patients [25]. Likewise, Zhu et al. identified stent dislodgement (OR 6.1, *p* = 0.01), urine immersion at the stent’s outer end (OR 5.5, *p* = 0.02), and less frequent stent replacement (every 6 vs. 3 months) as independent predictors of urinary tract infection in CU patients [66].

Collectively, current data show that the pyelonephritis rate of 22–33% in contemporary single-stoma CU is comparable to that of IC. These results may be due in part to technical refinements in modern techniques, which drive better stent-related outcomes and postoperative care.

### 6.3. Readmissions

Hospital readmission remains common after RC, regardless of approach or UD type, with 30- and 90-day rates from 18–25% and 17–39%, respectively [56,60,68,69]. Across the studies included in this review, readmission rates after single-stoma CU were generally similar to those observed with IC [29]. Thakker et al. found no significant differences in 30- or 90-day readmissions between CU and IC [8], while Fu et al. reported comparable 6-month rehospitalization rates among patients undergoing single-stoma CU, double-stoma CU, and IC (18.5, 22.2, and 22.2%, respectively, *p* = 0.9) [11]. Although not statistically significant, Tsaturyan et al. observed a lower readmission rate with their modified CU technique than with the standard Toyoda method (20.0% vs. 36.7%) [22]. While readmission rates appear largely similar between CU and IC, they may not fully capture differences in post-surgical recovery outcomes, as da Costa et al. found that CU patients spent more days alive and out of the hospital in the first 90 days after surgery than those with IC, and traditionally, patients undergoing CU have worse performance status. (75.4 ± 12.3 vs. 67.9 ± 14.7 days, *p* = 0.04) [29].

### 6.4. Urolithiasis

Urolithiasis is another significant long-term complication, with rates of 3.5–15.3% post-RC, and often requires standard urolithiasis management involving lithotripsy [32]. With an incidence of 17.4%, CU has been associated with this phenomenon, potentially related to impaired drainage from recurrent UTIs, chronic stenting, and stenosis [16,64]. However, recent series show improved outcomes for single-stoma CU, with Li et al. reporting lower urolithiasis rates with modified techniques compared with traditional techniques (12.2% vs. 40%) [25]. Postoperative weight changes may also influence stone formation after single-stoma CU, as Zhang et al. reported rates of 18.2% among patients with weight gain and 10.5% among those with weight loss [28].

### 6.5. Postoperative Renal Function

Historically, the development of postoperative renal failure has been a concern for patients undergoing CU, as long-term evidence has shown higher rates of this compared to other UD techniques [1]. However, direct comparison of different diversion types is complicated because patients with incontinent diversions and undergoing CU generally have worse baseline renal function, which could lead to selection bias and inconsistent results across different studies [70,71]. For example, Creta et al. evaluated patients ≥75 years old with high comorbidity burden (common underlying characteristics of patients selected for CU) undergoing RC with CU and observed a significant decline in median GFR from 74.3 to 54.6 mL/min/1.73 m^2^ at 6 months (*p* < 0.001), with progressive decline reaching 46.2 mL/min/1.73 m^2^ at 60 months [15]. On the other hand, a recent meta-analysis by Nabil et al. found no significant difference in renal deterioration between CU and IC (OR 0.81, 95% CI 0.39–1.68, *p* = 0.57) [72]. Instead, factors such as older age, lower preoperative eGFR, and postoperative hydronephrosis have been identified as independent predictors of high-grade chronic kidney disease after RC [70].

Single-stoma CU series generally report favorable renal outcomes, with minimal changes in GFR over time. For instance, Thakker et al. found no significant GFR difference between IC and single-stoma CU from pre-op to latest follow-up (0 [−12–10] vs. −4.5 [−7.5–15], *p* = 0.62) [8]. Similarly, Li et al. reported renal deterioration in 36% of patients overall, with a lower incidence after their modified single-stoma technique than with the Toyoda method (28.6% vs. 45.0%, *p* = 0.125) [25]. Current evidence suggests that modern single-stoma CU modifications have helped reduce rates of renal deterioration, although the retrospective nature of these findings should be taken into account. However, improved renal function helps maintain the perioperative benefits that make CU a preferred option for elderly and frail patients. Future long-term prospective studies could help clarify the benefits of single-stoma CU in preserving renal function, as the current literature lacks detailed long-term outcomes on this topic.

## 7. Quality of Life

Since the choice of UD may be heavily influenced by patient age, comorbidity burden, and functional status, the interpretation of quality-of-life (QoL) outcomes across diversion types requires careful consideration of the underlying patient characteristics and also makes their interpretation limited due to potential bias from them [42,71,73]. For example, Radu et al., using the EQ-5D-5L and EQ-VAS instruments, further highlighted the role of baseline functional status, reporting that patients requiring greater social support tended to have lower EQ-5D-5L index scores (*p* < 0.001), independent of diversion type [71]. In this context, interpreting QoL outcomes should consider both the UD type itself and the patients’ underlying demographic, social, and clinical characteristics.

Recent studies suggest that QoL outcomes for single-stoma CU may be closer to those of IC than to traditional bilateral CU. With a median follow-up of 26 months and using the EORTC-QLQ-C30 and FACT-Bl-Cys questionnaires (which assess domains such as global health status, functional health, symptom burden, body image, and satisfaction with urinary diversion), Arman et al. found that their modified single-stoma CU improved functional health, satisfaction with diversion, and global health status compared to standard double-stoma CU, with several domains nearing IC levels [23]. Despite the retrospective nature of their study and the small group sample sizes, Arman et al.’s findings suggest that single-stoma CU may yield better overall QoL outcomes and could be considered for more functional patients who are candidates for CU and have higher baseline activity. Similarly, both Longo et al. and Fuschi et al. reported comparable outcomes between single-stoma CU and IC across the urinary, bowel, and sexual domains, despite the CU group being older in the latter study [21,24]. Additional evidence indicates that patient-related characteristics after surgery and catheter dependence can significantly impact QoL after single-stoma CU. Zhang et al. found that postoperative BMI, late complications, and low catheter-free status were notably linked to worse global QoL and symptom scales following modified single-stoma CU [28].

Although QoL post-CU has traditionally been viewed as inferior to other diversion methods, modern single-stoma CU may offer outcomes comparable to IC in selected patients, as seen in Arman et al., especially when patient-related factors and common postoperative complications are minimized through technical improvements.

## 8. Conclusions and Future Directions

Cutaneous ureterostomy has re-emerged as an important urinary diversion option after radical cystectomy, particularly for elderly and frail patients for whom minimizing operative burden is a major concern. Available retrospective data indicate that modern single-stoma CU modifications have shown a trend towards shorter operative times and faster recovery compared to IC, while complication profiles, renal outcomes, and quality-of-life outcomes seem to be improved compared with historical CU data. Nevertheless, despite these improvements, stomal stenosis, catheter dependence, infectious complications, and long-term renal preservation will remain important considerations requiring ongoing surveillance. Importantly, randomized evidence is lacking, and most of the available data come from small retrospective studies with population overlap and potential selection bias, as elderly and frail patients are preferentially selected for CU. Care should be taken when interpreting these findings, as repeated reporting from the same populations and the retrospective nature of most of the included studies could unintentionally overemphasize certain findings. However, current evidence is promising and suggests that many historical limitations of cutaneous ureterostomy may potentially be mitigated through technical refinements and optimized perioperative care. Ultimately, future multicenter studies using propensity scores, or ideally clinical trials, with extended follow-up and standardized reporting of outcomes (such as catheter-free rates, renal function, and complication rates) are necessary to better understand the role of modern single-stoma CU in current bladder cancer treatment decisions, as most of the data presented in this manuscript comes from retrospective studies with small sample sizes.

## Figures and Tables

**Table 1:** Summarizes characteristics from studies evaluating the outcomes of single-stoma cutaneous ureterostomy.

							Peri-Operative Complications				Postoperative Complications						
Author Country	Study Design	Patient Population	Diversion Type	Number of Patients	Age (Yr)	Follow-Up Time (Mo)	Operative Time (min)	Mean EBL (mL)	Intra-OP Complications	LOS (Days)	30-Day Complications	90-Day Complications	Clavien-Dindo Grade ≥ 3	Stoma-Stenosis Rate		Stent-Free Rate	Post-OP Renal Function
**Longo et al.** **2016** **Italy** [21]	Single-center retrospective	Patients >75 yr with >2 ASA score	CU v. IC	70 patients (35 CU, 35 IC)	CU: 78.5 ± 2.1 IC: 78.8 ± 1.8	42.7 (12–72)	CU: 149.5 ± 35.1 IC: 225.8 ± 72.3	CU: 380 ± 93 IC: 510.5 ± 106.8	CU: 5.7% IC: 25.7%	CU: 8.8 ± 1 IC: 13.2 ± 1.7	CU: 28.5% IC: 57.1%	N/A	CU: 17% IC: 34%	N/A		N/A	N/A
**Tsaturyan et al.** **2019** **Armenia** [22]	Multicenter prospective	Males only	CU (Ariyoshi) v. CU (Yerevan)	52 patients (26 CU (Ariyoshi), 26 CU (Yerevan)	CU (Ariyoshi): 63.3 ± 6.7 CU (Yerevan): 62.8 ± 8.4	25.8 (1–37)	CU (Ariyoshi): 194.3 ± 24.7 CU (Yerevan): 201.7 ± 23.2	CU (Ariyoshi): 532.4 ± 125.8 CU (Yerevan): 510.8 ± 109	N/A	N/A	CU (Ariyoshi): 30% CU (Yerevan): 26.6%	CU (Ariyoshi): 43.3% CU (Yerevan): 26.6%	N/A	CU (Ariyoshi): 10% CU (Yerevan): 3.3%		CU (Ariyoshi): 42.3% CU (Yerevan): 76.9%	N/A
**Arman et al.** **2020** **Armenia** [23]	Multicenter prospective	Males only	CU (Ariyoshi) v. CU (Yerevan) v. IC	70 patients (25 CU (Ariyoshi), 23 CU (Yerevan), 22 IC)	CU (Ariyoshi): 61 (55–65) CU (Yerevan): 62 (57–68) IC: 57 (53–63)	26 (20–30)	N/A	CU (Ariyoshi): 450 (375–475) CU (Yerevan): 400 (300–450) IC: 400 (350–450)	N/A	N/A	CU (Ariyoshi): 20% CU (Yerevan): 13% IC: 22.7%	N/A	CU (Ariyoshi): 4% CU (Yerevan): 4.3% IC: 9.1%	N/A		N/A	N/A
**Fuschi et al. 2021** **Italy** [24]	Single-center retrospective	Patients >75 yr	CU v. IC	78 patients (41 CU, 37 IC)	CU: 82.4 ± 0.9 IC: 77.2 ± 0.9	14.4 (8–16)	CU: 186 ± 3.8 IC: 334 ± 4.1	N/A	N/A	CU: 6.5 ± 0.5 IC: 9.2 ± 0.6	CU: 37.4% IC: 57.6%	N/A	CU: 12.1% IC: 27.1%	N/A		N/A	CU Pre-OP GFR: 58.5 ± 4 CU 30-day GFR: 55.5 ± 3.7 IC Pre-OP GFR: 63.8 ± 4.2 IC 30-day GFR: 61.4 ± 3.8
**Fu et al.** **2023** **China** [11]	Single-center retrospective	All patients undergoing RC	CU v. DCU v. IC	108 patients (27 CU, 45 DCU, 36 IC)	CU: 71.9 ± 9.4 DCU: 70.1 ± 10.1 IC: 63.6 ± 7.3	24 (16–32)	CU: 240 (180–354) DCU: 246 (219–303) IC: 350 (302–446)	N/A	N/A	N/A	CU: 59.3% DCU: 71.1% IC: 61.1%	N/A	N/A	N/A		N/A	N/A
**Li et al.** **2023** **China** [25]	Single-center retrospective	Patients ≥70 yr with ≥3 ASA score	CU (Toyoda) v. CU (Li)	89 patients (40 CU (Toyoda), 49 CU (Li))	CU (Toyoda): 77.3 ± 4.1 CU (Li): 77.2 ± 4.5	44.1 ± 16.7	CU (Toyoda): 222.3 ± 36.3 CU (Li): 233.1 ± 29.7	CU (Toyoda): 225 (100–1600) CU (Li): 200 (100–1600)	N/A	CU (Toyoda): 16.4 ± 5 CU (Li): 17 ± 5.2	CU (Toyoda): 50% CU (Li) 1–2: 46.9%	N/A	CU (Toyoda) 3: 0 CU (Li) 3: 2%	CU (Toyoda): 37.5% CU (Li): 12.2%		CU (Toyoda): 71.8% CU (Li): 90.8%	Pre-OP GFR: CU (Toyoda): 62.8 ± 9.7 CU (Li): 61.2 ± 9.1 Last follow-up GFR: CU (Toyoda): 53.8 ± 12.5 CU (Li): 54.7 ± 11.8
**Nabavizadeh et al.** **2023** **United States** [26]	Single-center retrospective	All patients undergoing RC	Wallace anastomosis CU	31 patients	72.3 (67.7–77.7)	14.4 (7–25)	279 (214–334)	500 (300–900)	N/A	4 (4–7)	45.2%	54.8%	30-day: 45.2% 90-day: 54.8%	6.4%		93.5%	Pre-OP GFR: 49.5 (34–61.5) Post-OP GFR: 40 (32–50)
**Thakker et al.** **2023** **United States** [27]	Single-center retrospective	All patients undergoing RC	Modified Ariyoshi CU	28 patients	73 (66–78)	10 (7–14)	Open: 216 (192–240) Robotic: 306 (264–360)	N/A	N/A	3 (3–5)	74%	81%	29%	17%		74%	Pre-OP GFR ^a^: ≥60: 52% 30–59: 35% <30: 13% Post-OP GFR ^a^: ≥60: 48% 30–59: 35% <30: 13%
**Zhang et al.** **2023** **China** [28]	Single-center retrospective	Patients undergoing laparoscopic RC	CU v. IC	141 patients (65 CU, 76 IC)	CU: 70 ± 13.8 IC: 60 ± 13.7	12	N/A	N/A	N/A	N/A	N/A	Pyelonephritis: SCU 12.3% v. IC 13.1% Hydronephrosis: SCU 15.4% v. IC 22.4% Stone formation: SCU 18.5% v. 10.5% Stomal stricture: SCU 29.2% v. 11.8%	N/A	CU: 29.2% IC: 11.8%		CU: 72.3% IC: 89.5%	N/A
**Thakker et al.** **2024** **United States** [8]	Single-center retrospective	Patients undergoing open RC	CU (Ariyoshi) v. IC	56 patients (30 CU (Ariyoshi), 26 IC)	CU (Ariyoshi): 74 (70–78) IC: 71 (62–76)	15 (10–20)	CU (Ariyoshi): 216 (186–252) IC: 294 (270–366)	N/A	N/A	CU (Ariyoshi): 3 (3–6) IC: 4 (4–6)	CU (Ariyoshi): 67% IC: 61%	CU (Ariyoshi): 77% IC: 69%	CU (Ariyoshi): 46% IC: 77%	CU (Ariyoshi): 5% IC: 0		CU (Ariyoshi): 32% IC: 19%	Change in GFR from pre-OP to post-OP: CU (Ariyoshi): −4.5 (−7.5–15) IC: 0 (−12–10)
**da Costa et al.** **2026** **Brazil** [29]	Multicenter prospective	Patients ≥18	CU (Ariyoshi) v. IC	127 patients (57 CU (Ariyoshi), 70 IC)		CU (Ariyoshi): 71.5 ± 8.7 IC: 64.8 ± 9.3	12	Mean reduction of app. 1 h in CU (Ariyoshi) compared to IC group.	N/A	N/A	CU (Ariyoshi): 13 ± 8.9 IC: 20 ± 14.5	N/A	CU (Ariyoshi): 80% IC: 81%	CU (Ariyoshi): 28% IC: 41%	CU (Ariyoshi): 1.8% IC: 5.7%	N/A	N/A

CU: Cutaneous Ureterostomy; DCU: Dual stoma Cutaneous Ureterostomy; IC: Ileal Conduit; RC: Radical Cystectomy. ^a^: Values taken from the CU stent-free trial. Bolden outcomes reflect statistically significant results from the original studies.

**Table 2:** Evolution, modifications, and results of cutaneous ureterostomy.

Author Country	Year of Introduction	Significant Changes	Number of Patients Treated in Original Work	Results
**Simon et al.** **England** [30]	1852	First description of cutaneous ureterostomy being performed to treat a child with bladder exstrophy.	1	Ureteral patency was maintained for up to 12 months after the procedure.
**Ariyoshi et al.** **Japan** [34]	1975	Creation of a ureteral nipple with a triangular skin flap.	21	Of 26 patients, 65% of them developed a stable ureteral nipple and achieved a stent-free state.
**Toyoda** **Japan** [35,36]	1976	Creating a longitudinal ‘fish-mouth’ opening on the ureter and suturing it to the skin.	97 *	76% of 97 patients treated achieved tubeless state after a follow-up of 1 year. *
**Kerney et al.** **United States** [37]	1992	Use of a ‘horseshoe’ shaped skin flap to stabilize protruding stoma with transverse nephropexy to avoid ureteral retraction.	29	One third of patients preserved renal function from one to thirteen years postoperatively.
**Bolzano et al.** **Italy** [38]	2004	Ureters were covered by omentum and pulled through a cross-like fascial incision; ureters were then spatulated and everted in a ‘butterfly’ shape and fixed.	15	With a median follow-up of 15 (6–24) months, 14 of 15 (93%) patients with an ASA score of 3 were stent-free.
**Okada et al.** **Japan** [39]	2005	To prevent ureteral stenosis in the abdominal wall, a surgical stabilization step was introduced by fixating the anterior and posterior rectus sheaths. Ureters were then passed through the abdominal wall without being anchored and the stoma was created using the Toyoda method.	54	Of 59 renal units, 53 (89.8%) achieved a catheter-free state, improving from 60.5% of 43 renal units without this step.
**Tsaturyan et al.** **Arman** [22]	2019	The peri-ureteral parietal peritoneum is preserved and fixed to a tunnel formed by the anterior and posterior rectus muscle sheath; ureters are then anastomosed side by side on a single plate in a single oval-shaped stoma.	30	Compared to a control group without their modification, this technique achieved a catheter-free state in 79.2% (*n* = 20/26) vs. 42.3% (n = 11/26), with a median follow-up of 25.8 months.
**Li et al.** **China** [25]	2023	A 3 to 4 cm slit was made at each ureteral end. The ureters were then conjoined side by side using three interrupted sutures—one at the bottom of the slits and one on each side of the slits. Before bringing the ureters out of the abdominal wall, they reshaped and sutured the bilateral ureters together.	49	89 (90.8%) of 98 renal units with this technique and 56 (71.8%) of 78 without it remained catheter free after a mean follow-up of 44.1 ± 16.7 months.

* Data taken from the study by Terai et al. [36].

## Data Availability

No new data were created or analyzed in this study. Data sharing is not applicable to this article.
